# Lactoferrin in the Prevention and Treatment of Intestinal Inflammatory Pathologies Associated with Colorectal Cancer Development

**DOI:** 10.3390/cancers12123806

**Published:** 2020-12-17

**Authors:** Antimo Cutone, Giusi Ianiro, Maria Stefania Lepanto, Luigi Rosa, Piera Valenti, Maria Carmela Bonaccorsi di Patti, Giovanni Musci

**Affiliations:** 1 Department of Biosciences and Territory, University of Molise, 86090 Pesche, Italy; antimo.cutone@unimol.it (A.C.); g.ianiro@studenti.unimol.it (G.I.); 2 Department of Public Health and Infectious Diseases, University of Rome La Sapienza, 00185 Rome, Italy; mariastefania.lepanto@uniroma1.it (M.S.L.); luigi.rosa@uniroma1.it (L.R.); piera.valenti@uniroma1.it (P.V.); 3 Department of Biochemical Sciences, University of Rome La Sapienza, 00185 Rome, Italy

**Keywords:** lactoferrin, colorectal cancer, inflammatory bowel disease, ulcerative colitis, Crohn’s disease, microbial dysbiosis, intestinal barrier dysfunction

## Abstract

**Simple Summary:**

Colorectal cancer is the third most deadly and fourth most commonly diagnosed cancer in the world. Beside incorrect lifestyles, such as smoking or excessive consumption of red meat and alcohol, inflammatory bowel diseases are considered driving factors for colorectal cancer onset and development. It is known that chronic inflammatory processes can lead to both intestinal barrier disruption and perturbation of microbial flora, thus increasing cancer risk. To date, no treatment against these inflammatory pathologies has proved efficient and resolutive. The glycoprotein lactoferrin, a safe supplement for infant and adult foods, is involved in immune defense and endowed with a number of properties, including anti-microbial, anti-inflammatory and anti-cancer activities. This review outlines the most recent studies on lactoferrin as a potential candidate in the prevention and treatment of intestinal inflammatory pathologies that are associated with increased risk of colorectal cancer.

**Abstract:**

The connection between inflammation and cancer is well-established and supported by genetic, pharmacological and epidemiological data. The inflammatory bowel diseases (IBDs), including Crohn’s disease and ulcerative colitis, have been described as important promoters for colorectal cancer development. Risk factors include environmental and food-borne mutagens, dysbalance of intestinal microbiome composition and chronic intestinal inflammation, with loss of intestinal epithelial barrier and enhanced cell proliferation rate. Therapies aimed at shutting down mucosal inflammatory response represent the foundation for IBDs treatment. However, when applied for long periods, they can alter the immune system and promote microbiome dysbiosis and carcinogenesis. Therefore, it is imperative to find new safe substances acting as both potent anti-inflammatory and anti-pathogen agents. Lactoferrin (Lf), an iron-binding glycoprotein essential in innate immunity, is generally recognized as safe and used as food supplement due to its multifunctionality. Lf possesses a wide range of immunomodulatory and anti-inflammatory properties against different aseptic and septic inflammatory pathologies, including IBDs. Moreover, Lf exerts anti-adhesive, anti-invasive and anti-survival activities against several microbial pathogens that colonize intestinal mucosa of IBDs patients. This review focuses on those activities of Lf potentially useful for the prevention/treatment of intestinal inflammatory pathologies associated with colorectal cancer development.

## 1. Introduction

Lactoferrin (Lf) was first fractionated as an unknown ‘‘red fraction’’ from cow’s milk in 1939 [1]. Only in 1960, the red protein from both human and bovine milk was defined as a transferrin-like glycoprotein [2,3]. Lf is present in the milk from different species in a lactation stage-dependent manner, with higher concentrations in colostrum and lower content during the middle and late stages of milk maturation [4]. Lf is also produced by exocrine glands and is released in biological fluids such as mucus, seminal fluid, tears, saliva, bronchial secretion, bile, gastrointestinal fluids, urine, blood plasma and amniotic fluid [5]. In the hematopoietic system, Lf is expressed during the myelocyte maturation stage, then it is stored in the secondary granules of neutrophils [6,7] and finally released at infection and inflammation sites. As a matter of fact, Lf exerts many protective functions including anti-bacterial, anti-fungal, anti-parasitic and anti-viral ones [8,9,10,11] as well as homeostatic roles including immunomodulatory, anti-inflammatory, antioxidant and anti-cancer activities [12,13,14,15]. Lf is also emerging as an essential regulator of cellular and systemic iron homeostasis [16,17].

Numerous structural studies establish that Lf is folded in two homologous lobes (N- and C-lobe), sharing about 40% sequence identity, connected by a three-turn helix. Each lobe presents four conserved residues (Y92, Y192, H253, D60 for N-lobe and Y433, Y526, H595, D393 for the C-lobe), responsible, together with two CO_3_^2−^ ions, for the binding of a ferric iron (Fe^3+^) with high affinity (Kd = 10^−20^ M) [18]. By virtue of its iron-binding ability, Lf can switch between two conformational states, the open iron-free form (apo Lf) and the closed iron-bound form (holo Lf) [19]. Under physiological conditions, native Lf presents a 15–20% iron saturation rate, characterized by the prevalence of apo and monoferric Lf. On the other hand, in infected and inflamed sites, characterized by higher concentrations of free iron, the holo-form prevails. In this way, Lf reduces free iron-induced reactive oxygen species (ROS) formation and radical damage as well as iron availability to pathogens and cancer cells [15,20]. Additionally, holo Lf shows higher temperature stability and resistance to proteolytic digestion with respect to the apo form [21]. Thus, it is of utmost importance to consider Lf iron saturation rate when carrying out experiments as it can influence Lf functioning [22,23]. Lf is a cationic protein (pI ca. 9) with high prevalence of basic residues at the N-terminal region. To date, two major peptides, derived from the Lf N-terminal tryptic digestion, have been described, Lactoferricin (Lfcin) and Lactoferrampin (Lfampin). These positively charged peptides, physiologically involved in gut homeostasis, are endowed with numerous biological functions, including anti-microbial and anti-cancer ones [24,25]. Indeed, due to their positive charge, Lfcin and Lfampin can interact with the negatively charged surface of pathogens or cancer cells, thus inducing cell lysis [26,27]. Of note, a role of Lf and Lfcin as probiotic components, by promoting the growth of certain beneficial strains, including Bifidobacteria and Lactobacilli, has been reported [28,29].

Human (hLf) and bovine Lf (bLf), despite bearing different glycosylation sites (Asn 138 and Asn 479 in hLf; Asn233, Asn368, Asn476, and Asn545 in bLf) [30,31,32], have high sequence similarities and homologous functions [18]. BLf is classified as a “generally recognized as safe” (GRAS) substance by the USA Food and Drug Administration (FDA). Therefore, most of the in vitro [33,34] and in vivo [35,36] experiments as well as clinical trials [37,38] have been so far conducted using commercial bLf.

Lf availability is strictly dependent on age and individual dietary habits. In the lactation stage Lf intestinal absorption is obviously guaranteed by maternal milk consumption, on the other hand the “normal” diet is a poor source of exogenous Lf, whereas endogenous Lf is exclusively guaranteed by neutrophils degranulation at infection and inflammation sites. Interestingly, human milk-derived and neutrophil-derived Lf apparently differ only in the composition of their carbohydrate side chains; human milk-derived Lf contains fucose residues whereas neutrophil-derived Lf does not [39,40]. Functionally, both types of Lfs appear to be equivalent [41,42] and no reports have investigated how the carbohydrate side chains and/or their composition contribute to its potential immunogenicity.

Although it has a high molecular mass, ca. 80 kDa, milk-derivative Lf is readily absorbed by infants due to their freely permeable gut [43]. Moreover, moderately high pH and low secretion of proteolytic enzymes, characterizing infant gastrointestinal system, allow intact Lf to reach the intestine and interact with its specific receptors [44]. Lf in human infant supports ca. 50% of iron absorption, whereas it engages ca. 5% and 20% of iron from bovine milk and infant formula, respectively [45]. In contrast, adult oral consumption of recombinant hLf from transgenic cow milk results in its complete digestion in the upper gastrointestinal tract [46], while only 40% of bLf is degraded [47]. Surprisingly, it appears that, in human adults, bLf is more resistant to proteolysis than hLf, suggesting that hLf is “designed” to be ingested orally only during infancy. In this regard, bLf oral administration should be preferentially performed before meals, in order to avoid its digestion by gastric proteases, activated after meal consumption [48].

Specific studies on hLf bioavailability have identified peculiar hLf receptors (hLfRs) on the apical side of enterocytes, responsible for its internalization and subsequent vehiculation into the bloodstream and delivery to tissues [49,50]. Intelectin-1 (ITLN-1) is a high affinity receptor for Lf (Kd = 10^−6^ M) [51,52], able to transduce several Lf-mediated functions, including facilitation of intestinal iron absorption in infants [53,54] and immune boosting [55,56]. In humans, ITLN-1 is expressed in the intestinal brush-border, Paneth and goblet cells [44] as well as on biliary epithelium [57]. Moreover, Lf internalization in hepatocytes from plasma [58] and translocation across the blood-brain barrier (BBB) [59] was demonstrated to be mediated by LDL receptor protein 1 (LRP1). Additionally, the Lf binding receptor CD14 was exclusively found on monocytes, nucleolin on lymphocytes and asialoglycoprotein receptor (ASGPR) in the liver [44,60].

Lf exploits this wide range of receptors to activate different cellular responses, either by triggering intracellular signaling [61] or by direct cell entrance through clathrin-mediated endocytosis and subsequent translocation into the nucleus [62,63]. Once in the nucleus, Lf can modulate gene expression, thus exerting its anti-inflammatory and anti-cancer activities.

The heterologous interaction between bLf and hLfRs was first demonstrated only in 2008 [64], as well as the ability of bLf to reach the cell nucleus in human cells [23,65], thus partially elucidating the molecular mechanism by which hLf and bLf are able to exert similar functions in humans. Indeed, bLf is currently recognized as a bioequivalent of hLf.

To date, bLf is principally used in infant formula to improve iron absorption and the immune response in newborn and as supplement in some food preparations. However, the optimization and the efficacy of bLf supplemented products are still under debate as bLf bioavailability strictly depends on the source and the methods for industrial production, which can influence its purity, integrity, stability and, consequently, its activity [21].

## 2. Lactoferrin in Natural and Supplemented Foods

**Figure 1:**
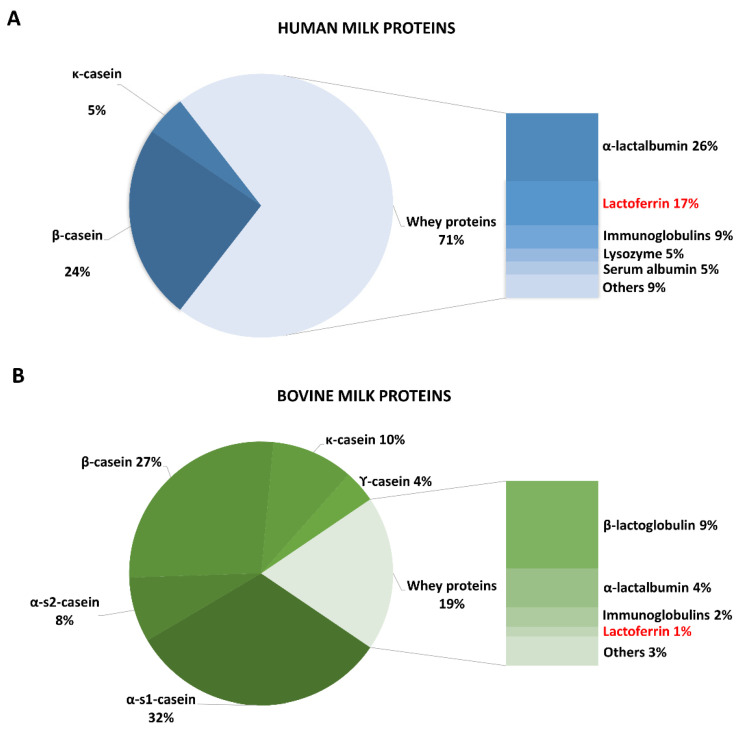
Percentage of the main proteins in human (**A**) and bovine (**B**) milk.

Infants and adults can be exposed to endogenous, neutrophil-derived Lf, but the first exposure to exogenous Lf is through consumption of breast milk and/or milk-based infant formula. Milk proteins consist of two main components, caseins (CNs) and whey proteins. While human milk is characterized by 71% of whey proteins and 29% of CNs [66,67], in cow’s milk CNs accounts for 81% of total proteins, and whey proteins only for 19% [66,67,68] (Figure 1A,B).

CNs are found in milk as micelles, in which salt bridges between calcium phosphate and residues of β- and α-caseins are particularly important to maintain the particles structure [69]. Overall, casein micelles exert immuno-modulatory functions, repress cell growth and reduce inflammation [70], and also provide adequate nutrients to the neonate and prevent pathological calcification or amyloidosis [71].

Whey proteins, including Lf, constitute a class of proteins exerting different functions. α-lactalbumin, the predominant whey protein in human milk, is mainly involved in the biosynthesis of lactose [72], but also shows anti-carcinogenic [73] and anti-microbial [74] activities. β-lactoglobulin, the most abundant whey protein in bovine milk (absent in human milk), enters the upper intestine in intact form [75], thus possibly triggering an allergic response in a minority of infants. In addition, serum albumin, which accounts for ca. 5% of human milk proteins, is a source of amino acids for the breast-fed infant. Interestingly, high concentrations of whey proteins involved in boosting mucosal immune system, such as Lf and Immunoglobulins, are predominantly present only in human milk, thus contributing to infant’s defense against infections. The total amounts of these components in bovine milk are much lower than in human milk (Figure 1A,B), and significantly minimized or absent in infant formula. This could partly account for the increased incidence of infections in formula-fed infants, compared with breast-fed infants [76], thus supporting supplementation of formulas with these compounds.

In humans, the mean levels of Lf are approximately 5 mg/mL in colostrum and 1 mg/mL in mature milk [66,67,77], whereas cow milk contains approximately 0.1 to 0.2 mg/mL of Lf [66,67,68]. Curiously, levels of Lf are much higher in human than in bovine milk, representing 17% vs. 1% of total proteins, respectively [66,67,68] (Figure 1A,B).

Lf bioavailability relies exclusively on exogenous source from diet, as endogenous Lf, in physiological condition, is synthetized and secreted only by exocrine glands, thus not significantly influencing systemic Lf concentrations. On the other hand, several in vivo studies, carried out in adult pigs [78], rats [79] and mice [49], have shown that exogenous Lf is vehiculated into systemic circulation via both the lymphatic pathway and the portal vein. Therefore, to ensure Lf bioavailability, a relevant consumption of Lf-bearing foods is needed. However, these are limited to milk and dairy products when natural foods are considered. Worldwide, cow milk is the most commonly consumed milk, representing 81% of the world milk production, followed by milk from other species such as buffalo (15%), goat (2%), sheep (1%) and camel (0.5%) [80]. Provision of active Lf from these milks and their derivatives is influenced by different factors, including milk origin and industrial procedures. First, the composition and concentration of different milk constituents can vary depending on several genetic and environmental factors, including lactation stage, breastfeeding routine, parity, age and other maternal characteristics such as regional differences and, in some situations, season of the year and maternal diet [81]. Moreover, during manufacture of Lf-containing foods, it is critical to preserve the protein structure/integrity from the raw materials to the finished product. The major unit operations in any food processing operation include formulation, application of acid, chemicals and heat treatment or use of other technologies, such as high pressure processing, with final packaging and storage. In this regard, although new technologies have been employed in the production of dairy foods to maintain Lf biological properties [82], they remain a poor/modest source of exogenous Lf (Table 1).

To overcome the intrinsic limitations due to modest Lf concentration in milk and natural dairy products, companies have focused their efforts on the use of exogenous Lf to supplement foods.

The use of human Lf in recombinant form produced by different systems, including plants such as rice [91], yeast such as *Pichia pastoris* [92], fungi such as *Aspergillus awamori* [93] and transgenic cattle [94], implies high industrial costs. Moreover, functional properties of recombinant hLf can differ from the natural native glycoprotein, due to differences in iron-saturation rate, glycosylation patterns, purity and stability. Additionally, the consumer’s preference towards genetically modified food remains very low.

For these reasons, milk-derivative bLf, due to its high grade of bioequivalence with the human counterpart, has been largely employed as the major source of exogenous Lf for food supplementation. BLf can be easily isolated from cow’s milk by cation-exchange chromatography, currently used for large-scale production by manufacturing companies. At present, bLf is produced and commercialized by many companies with a worldwide production scale increased up to 300 k tons with a market size estimated at USD 203.7 million in 2019 [95].

Currently, several bLF-supplemented foods are commercially available, including infant formula, yogurt, powdered and skim milk, milk-type drinks and desserts, supplemental tablets and chewing gum (Table 2).

The beneficial effects of these bLf-containing products on health have been proved in clinical and animal studies. Studies on the effects of bLf-supplemented infant formulas were the very first reported: these studies documented an improvement of intestinal microbial flora [98,99], an increase of serum ferritin [100] and hematocrit [101] levels, and reduced lower respiratory tract illnesses [101]. Moreover, the effect of bLf-supplemented yogurt on rotaviral gastroenteritis and the effect of tablets on chronic hepatitis C (CHC) and *Helicobacter pylori* gastric infection [102,103,104] were reported.

In particular, children treated with bLf-supplemented yogurt showed a reduction in the frequency and duration of both vomiting and diarrhea following rotavirus infection [102], whereas oral administration of bLf tablets was effective in increasing Th1-cytokine dominant environment in the peripheral blood of CHC patients, thus possibly favoring IFN-mediated eradication of HCV [103], as well as the suppression of *H. pylori* colonization [104].

Globally, studies on the beneficial effects of orally administered bLf on intestinal flora [105] and immune system [53] as well as against infections [106,107,108,109], inflammation [14,20] and cancers [15,110] strongly support the usefulness of bLf-supplemented foods.

## 3. Inflammatory Bowel Diseases (IBDs) and Colorectal Cancer Development

Inflammatory bowel diseases (IBDs) are chronic inflammatory and relapsing conditions of the gastrointestinal tract [111]. IBDs, whose incidence and prevalence are increasing since the second half of the 20th century, occur in both males and females and are increasingly diagnosed among older people [111,112,113].

The common types of IBDs are represented by Crohn’s disease (CD), with an inflammation affecting any area of the gastrointestinal tract but most commonly the terminal ileum or the perianal region, and Ulcerative Colitis (UC), with an inflammation that usually affects the colon and the rectum [114]. In particular, CD displays a thickened sub-mucosa, transmural inflammation, fissuring ulceration and granulomas, and it is frequently associated with complications such as abscesses, fistulas and strictures [114]. Conversely, UC shows a non-transmural inflammation limited to the mucosa and submucosa with cryptitis and crypt abscesses [114].

The etiology of IBDs is not yet fully clarified, but it is generally accepted that the chronic inflammation, in genetically susceptible subjects, is related to (i) external environment, (ii) intestinal microbial flora, and (iii) inappropriate immune response against commensal gut microbiota [111]. In addition, these pathologies are characterized by a loss of integrity of the intestinal barrier, with an increase in epithelial damage, mucosal permeability and host susceptibility to pathogens [115,116].

In healthy subjects, gut microbiota is 90% composed of Bacteroidetes and Firmicutes, with the remaining fraction belonging to rare phyla such as Proteobacteria and Actinobacteria as well as fungi, viruses and protists [117]. On the other hand, IBDs subjects present an intestinal microbiome dysbiosis, characterized by an increase in the number of mucosa-associated bacteria and a reduction in the overall biodiversity, with the decrease in beneficial bacteria such as Bacteroidetes and Firmicutes, and a relative increase in Proteobacteria, especially Enterobacteriaceae [114,118,119]. In particular, in UC patients a reduction is observed in the beneficial *Faecalibacterium prausnitzii* [120], able to induce the production of the anti-inflammatory cytokine interleukin (IL)-10 and to inhibit the synthesis of some pro-inflammatory cytokines, such as IL-12 and interferon (IFN)-γ [121]. Moreover, a relevant increase in Proteobacteria is observed, especially in CD patients [117,118]. In particular, *Pseudomonas* spp., *Listeria monocytogenes*, *Yersinia* spp. as well as adhesive-invasive *Escherichia coli* (AIEC) have been proposed as triggers of CD [117,118,122].

Other than bacteria, fungi have been reported to have a potential role in the pathogenesis of IBDs [123]. Although representing a minor component of the gut microbiota, the fungal microbiome (also called mycobiome) is recently emerging as a key player in maintaining intestinal homeostasis and health, and its alterations can result in the onset and development of various diseases [124]. Mycobiome of healthy subjects is mainly constituted by fungi belonging to the genera *Candida*, *Cryptococcus*, *Malassezia*, *Aspergillus*, *Saccharomyces*, *Galactomyces*, *Trichosporon*, and *Cladosporium*, with *C. albicans, S. cerevisiae* and *M. restricta* being the most common species [125]. The tight interplay between fungi and bacteria is now emerging as a fundamental element in the maintenance of gut homeostasis. Commensal fungi, such as *S. cerevisiae* and *C. albicans*, have been found to buffer bacterial dysbiosis following antibiotic treatments as well as to protect the host against colitis by ameliorating mucosal tissue injury and immune imbalance [126]. On the other hand, in a murine model of dextran sulphate sodium (DSS)–induced colitis, alteration of mycobiome following anti-fungal treatment resulted in the concurrent increase of pathogenic bacteria, compared to mice treated with antibiotics, thus exacerbating disease severity [127]. Fungi can also modulate intestinal microbiota, as shown by the administration of small amounts of *C. albicans* to mice after antibiotic treatment causing significant alterations in the gut microbiota, from phyla to family-level, which are irreversible in the long-term [128].

The alteration of mycobiome has been causally associated to IBDs in different studies. In CD patients an increase in *S. cerevisiae, C. albicans, Clavispora lusitaniae, Cyberlindnera jadinii* and *Kluyveromyces marxianus*, associated to bacterial dysbiosis, has been reported [129]. Moreover, the profile analysis of fungal microbiota in IBD (vs healthy) subjects highlighted a spike in Basidiomycota/Ascomycota ratio with an increased proportion of *C. albicans* and *M. sympodialis* and a decrease of *S. cerevisiae* [130]. These changes in mycobiome composition in CD patients result in intestinal mucosal inflammation as demonstrated by Li and colleagues, which have found an increase in *Alternaria brassicicola*, *Gibberella moniliformis*, *C. neoformans* and *Candida* spp. colonization, with the concurrent up-regulation of IFN-γ and TNF-α [131]. A dysbalance in mycobiota can also activate host innate immune response against the fungal cell wall components, such as β-glucans, mannans and chitin, which can trigger several immune cell receptors, including toll-like receptors, dectin-1, members of the scavenger receptor family (SCARF1, CD5 and CD36) and components of the complement system [123]. Based on these novel findings, it is conceivable that IBDs pathogenesis could be linked to the loss of the fine interkingdom interaction between bacterial and fungal flora. Hence, novel IBD treatments should be aimed not only at counteracting bacterial dysbiosis but also at restoring the complex interplay between bacteria and mycobiota, thus globally rebalancing gut homeostasis.

Lately, with the advent of next-generation sequencing, a potential role in IBDs pathogenesis was assigned to the most abundant entity constituting the gut microbiota: viruses. The entire population of bacterial and eukaryotic viruses was termed as the human virome. Among them, bacterial viruses, the bacteriophages, populate the gut 10 times as many bacteria [132], thus supporting a potential role for them in the maintenance of bacterial flora diversity and balance. To date, few studies have suggested a causative role of gut virome dysbiosis to IBD. The study by Perez-Brocal and colleagues showed a significant increase in *Alteromonadales*- and *Clostridiales*-infecting phages, with a concomitant abundance in *Retroviridae* family, in subjects with CD compared to healthy ones [133]. A recent study reported a *Caudovirales* bacteriophage expansion in UC subjects, associated to a decrease of their richness and diversity, and significantly correlated to intestinal inflammation [134]. Interestingly, the putative causative role of bacteriophages in IBD pathogenesis was demonstrated in germ-free mice challenged with increasing levels of *Lactobacillus-*, *Escherichia-* and *Bacteroides*-infecting phages, resulting in the alteration of mucosal immunity and induction of colitis due to triggering of TLR9 signaling induced by IFN-γ production [135].

The use of bacteriophages as therapeutic agents in IBDs-associated microbial dysbiosis has also been proposed. A single phage cocktail administration to DSS-treated mice was reported to reduce colitis severity by directly decreasing fecal AIEC number and the adherent flora [136]. Of note, bacteriophage treatment against AIEC in CD patients is currently under investigation in a clinical trial (www.clinicaltrials.gov; NCT03808103).

Despite the predominance of bacterial viruses, the human gut virome is characterized by a higher diversity in eukaryotic viruses among individuals. Eukaryotic viruses colonize the intestinal mucosa since the first years of age. They belong to *Adenoviridae*, *Anelloviridae*, *Astroviridae*, *Parvoviridae*, *Picornaviridae* and *Picobirnaviridae* families and their diversity and richness vary according to environmental exposure [137]. Only few studies have reported an association between viral dysbiosis and IBDs [134,138,139] and it is still unclear if eukaryotic viruses are the triggers or simple bystanders of the disease. Most of these viruses remains latent for the entire lifespan in healthy people, whereas they can reactivate in IBD patients due to stress conditions. The only report on a mechanistic role of eukaryotic viruses in IBD described *Norovirus* as a potent agent able to exacerbate intestinal inflammation in transgenic murine models of spontaneous colitis [140,141]. Therefore, new studies are needed to unveil the possible role of eukaryotic viruses in IBD onset and development.

Regarding protozoa, their role in gut homeostasis is still under debate, and very few studies have been conducted to ascertain their impact on IBDs. In particular, although *Blastocystis* spp. has been associated to an increase of diversity in the human gut microbiota [142], recent studies have highlighted a link between *B. hominis* infection and UC activation and persistence [143,144]. Hence, further comprehensive studies will be required to unveil the actual role of protozoa on IBDs.

Large scale alteration in the gut microbiota, primed on one or more of its entities, can trigger an altered intestinal immune response in the gut mucosa, with a decrease of commensals endowed with anti-inflammatory properties and an increase of those able to trigger an inflammatory response, overall leading to a severe inflammatory state [117,118]. The analysis of inflamed mucosa of IBDs patients has revealed an increased expression of pro-inflammatory cytokines such as IL-1, IL-6, IL-8, IL-12 and tumor necrosis factor (TNF)-α [117,145,146,147]. In particular, IL-6, whose levels are correlated with the severity of disease, is highly expressed in both serum and tissue of IBDs patients and plays a central role in several immune responses [147,148,149]. Of note, IL-6 can lead to nuclear factor (NF)-kB activation and NF-kB nuclear translocation, inducing the expression of intercellular adhesion molecule 1, an important adhesion molecule in the neutrophil-epithelial interactions in IBDs [117,150]. Furthermore, IL-6 prevents apoptosis with a consequent T-cell accumulation leading to chronic inflammation [151]. Overall, IL-6 shows a key role in immune response and a dysregulation of its pathways can contribute to the progression of chronic disease [152]. Patients suffering from IBDs also show an increase in neutrophils and macrophages infiltrates as well as in ROS [153,154].

A dysbalance of secretory immunoglobulins (Ig) in IBDs has also been reported [155,156]. Several studies have shown that both IgA- or IgG-coated bacteria increase in feces of active IBD patients [157,158], and that the amount of IgA and IgG is positively related to the degree of disease activity [159]. However, the functional implications of this immune activation remain largely unknown, as it is widely believed that B cells are not involved in IBD pathogenesis. Of note, a recent study highlighted the involvement of the antibody receptor Fcγ (FcγR) in the IgG-related inflammatory response in UC patients. The authors reported an acute induction of anti-commensal IgGs with a concurrent up-regulation of FcγR signaling, which in turn activates NLRP3- and ROS-dependent production of both IL-1β and neutrophil-recruiting chemokines [160].

Altogether, intestinal dysbiosis, abnormal gut colonization and inappropriate immune response against commensal gut microbiota lead to chronic destructive inflammation and consequent epithelial damage.

Chronic infection and the persistence/severity of bowel inflammation can lead to colorectal dysplasia and eventually to colorectal cancer (CRC) [161]. IBDs are high-risk conditions for CRC [162], and IBD patients are 2–6 times more likely to develop CRC and develop it predominantly at young age [163]. The incidence of CRC in IBDs patients is about 2% 10 years after the onset of the disease, 8% after 20 years, and 18% after 30 years [161]. CD–associated CRC is observed in a similar time frame as in UC [164,165,166], but UC–associated CRC is most common in rectum and sigmoid colon, whereas CD–associated CRC is evenly distributed between the different colon segments [161,167]. Differently from sporadic CRC (SCC), where CRC begins with adenoma formation that progresses over time to advanced adenomas with high grade dysplasia and carcinoma, IBDs-associated CRC is developed following persistent inflammation and regeneration leading to dysplasia and carcinoma [162]. IBDs patients are predisposed to developing a form of CRC, known as colitis-associated CRC (CAC) [151]. CAC affects young people and progresses from flat and non-polypoid dysplasia to invasive adenocarcinoma more frequently than SCC [168]. Moreover, differently from SCC, where dysplastic lesions are localized in one or two focal areas of the colon, in CAC dysplasia or cancer can be multifocal [168]. CD subjects have approximately 1.8 times the risk of developing CAC, while the risk for subjects with UC is up to 8 times higher compared to the general population [151].

The cancer risk factors include a family history of CRC, the duration of IBD, and the severity and persistence of inflammation [163]. However, some studies have pointed out how CRC risk does not correlate with IBDs activity: patients with a quiescent disease present a similar risk of developing CRC to those who have an active disease [164,169]. Indeed, even the chronic low-grade inflammation, common in patients with IBD in clinical remission, keeps high the risk of clinical relapse, hospitalization, surgery, and CRC [170]. Therefore, it appears that it is more the chronicity than the degree of the intestinal inflammation which plays a major role in CRC development. For example, prolonged IL-6 signaling can (i) activate STAT3, a major oncogenic transcription factor [171,172], (ii) down-regulate the tumor suppressor p53, and (iii) up-regulate the oncogene c-myc in colon epithelial cells [173]. In addition, chronic inflammation can cause oxidative DNA damage, which can result in neoplasia and cancer development when involving key growth regulatory genes [154,161,174]. ROS can alter the regulation of genes that encode anti-carcinogenesis factors, such as DNA mismatch repair proteins and p53, as well as transcription factors, such as NF-kB [175]. Of note, loss-of-function mutations in p53 that occur in IBDs-associated neoplasia [163,168] exacerbate the already inflamed IBD mucosa accelerating the development of carcinoma [176]. Moreover, in IBDs-associated CRC, although less frequently, induction of the K-ras oncogene can also occur [161], and mutations in APC can arise in the late stage of the dysplasia-carcinoma sequence where they promote NF-kB mediated cytokine secretion, neovascularization, and maintenance of tumor growth [177]. Finally, recent studies have demonstrated that patients with IBDs-associated CRC have a higher risk of death (52%) compared to those without IBDs [162,178].

Currently, no efficient therapy for IBDs resolution has been found yet. Most of the approaches rely exclusively on targeting microbiome dysbiosis or inflammatory processes. Conventional therapies aimed at rebalancing the intestinal microbiota include prebiotics and probiotics or fecal microbiota transplantation (FMT) [179]. Other therapies include immunosuppressors, such as 5-aminosalicylic acid, glucocorticosteroids, thiopurines and methotrexate, monoclonal antibodies directed against pro-inflammatory cytokines such as TNF-α, IL-12, IL-23, or anti-homing antibodies directed against α4β7 integrin [179]. However, prolonged therapies altering the immune system may promote microbiome dysbiosis and carcinogenesis.

In this context, Lf could represent a very promising tool able to act on multiple aspects of IBDs pathogenesis, including microbiota dysbiosis and destructive chronic inflammation, without inducing significant adverse effects, even at high dosage.

### 3.1. Lactoferrin Anti-Microbial Activity against IBDs

The anti-microbial activity has been described as the very first Lf function, linked to the ancestral host defense mechanisms to combat pathogens infections. This activity, evaluated in several in vitro [34,180,181,182] and in vivo [35,36,183] models, can be both dependent or independent of Lf iron binding ability [20,184]. Through its capability to chelate two ferric ions per molecule, Lf exerts a bacteriostatic action limiting microbial growth in infection sites by producing an iron-deficient environment [20]. On the other hand, Lf is able to exert a microbicide activity independently of its iron-chelation ability. Lf-mediated bacterial lysis, that appears to be similar for both Gram-negative and Gram-positive bacteria, results from the perturbation of bacterial membranes. In Gram-positive bacteria, the bactericidal activity of Lf is mediated by electrostatic interactions between the negatively charged lipid layer and the positively charged Lf surface, causing changes in the membrane permeability [20]. In Gram-negative bacteria, cellular lysis is performed through (i) direct interaction of Lf with lipopolysaccharide (LPS) [185,186,187] and (ii) Lf ability to sequester Ca(II) thus inducing the release of LPS [188]. Moreover, independent of iron-chelation, Lf is also able to inhibit: (i) bacterial adhesion and invasion through its competitive binding with surface components of host cells and/or bacteria, thus decreasing bacterial–host cell interaction and bacterial internalization [34,189,190,191,192], (ii) bacterial intracellular survival through still unknown mechanisms [34,180] and (iii) biofilm formation [193].

The anti-microbial activity of Lf has been demonstrated towards several bacteria involved in infectious processes, including *E. coli*, *Salmonella typhimurium*, *Shigella dysenteriae*, *L. monocytogenes*, *Streptococcus* spp., *Staphylococcus* spp. [184]. On the other hand, Lf has been shown to promote the growth of some beneficial bacteria, such as Lactobacillus and Bifidobacteria [184], that make an important contribution in maintaining the integrity of the gut epithelial barrier and reducing the adherence or translocation of enteric pathogenic bacteria [105]. The importance of Lf in the development of beneficial bacteria is confirmed by in vivo studies on neonates and toddlers, demonstrating that high levels of fecal Lf contributed to the initiation, development, and composition of the neonatal gut microbiota [194] as well as to early host-microbe interaction [195]. Moreover, Lf contributes to the formation of the rich intestinal microbiota diversity [105,106] that is crucial and protective for sustaining a microbial equilibrium and for the integrity of the mucosal barrier [196].

It is well known that the gut microbiota provides benefits to host’s physiology, including protection from pathogens, nutrition, metabolism, and immune system [118,196]. Commensal and symbiotic bacteria improve mucosal ability to absorb food, as well as to control the colonization by pathogenic microorganisms through competition for nutrients and stimulation of basal immune responses [196,197,198,199]. However, in some pathologies, such as IBDs, a reduction in gut microbial diversity can occur [200,201]. In particular, in biopsy specimens obtained from CD patients *Pseudomonas* spp. is significantly more prevalent compared to specimens from non-CD patients [122] and AIEC is abnormally predominant in early and chronic ileal lesions of CD patients [202]. In addition to these bacteria, *L. monocytogenes* and *Yersinia* spp. have also been proposed as triggers of CD [117,118]. Furthermore, some commensal microorganisms, defined pathobionts, can shift towards a pathogenic phenotype in IBDs patients [200,201]. AIEC strains, implicated in the etiology of IBDs, are considered pathobionts because they promote inflammatory diseases due to the adaptive evolution of their genome in a specific and susceptible host [201,203,204,205,206]. These bacteria are able to adhere to, invade and persist into intestinal epithelial cells (IECs) and macrophages, and are also able to block the host cell cycle in S phase by inducing DNA damage [202,207,208,209,210].

Several studies showed that Lf is capable of exerting anti-microbial activity against these bacteria [36,180,211,212,213,214]. Indeed, in vitro studies have demonstrated that Lf acts against *P. aeruginosa* planktonic and biofilm-forming cells by depriving the environment of iron, thus reducing bacterial growth and colonization, and by permeabilizing bacterial membranes thus leading to bacterial lysis [36,212,214,215]. Moreover, several studies have shown that Lf exerts anti-microbial activity also against *Yersinia* spp. and *L. monocytogenes* through the binding of Lf to both bacterial adhesins and host cells [20,213,216]. Notably, it has been demonstrated that Lf significantly inhibits Yersinia entry into IECs by competitively binding to cell surface glycosaminoglycans (GAGs), known mediators of bacterial adhesion to host cells [216].

Lf has proven to be a glycoprotein capable of exerting an anti-microbial activity also against *E. coli* LF82 [34,180], a prototype of AIEC strains. The study conducted in 2014 on a IEC model showed that bLf induces a significant decrease of AIEC LF82 invasion ability as well as of its intracellular survival [180]. These results have been confirmed by a study in 2019, showing that a 12 h pre-treatment of IECs with bLf significantly decreases LF82 invasion and bacterial intracellular survival, these effects being still present when cells were infected 10 h after bLf removal from the culture medium [34]. This activity was found to be independent of the modulation of CEACAM-6 receptor, the main cell surface molecule for AIEC adhesion, as well as from apoptotic process. Furthermore, the authors showed that bLf was able to protect host cells from LF82-mediated DNA damage [34].

As mentioned above, despite most of our knowledge on IBD pathogenesis relies on bacterial dysbiosis, mycobiome is emerging as a pivotal actor in the onset and development of the disease. In this respect, Lf could be considered a good candidate for IBDs treatment due to its anti-fungal activity. Most of the studies have been carried out on members of the *Candida* spp., one of the main fungi involved in IBDs, with *C. albicans, C. tropicalis* and *C. krusei* showing the highest susceptibility to Lf treatment [217,218]. As with bacteriostatic activity, the antifungal effect of Lf was firstly attributed to its iron-binding ability [219], since growth of both *Candida* spp. and *Cryptococcus* spp. was restored by iron supplementation of the medium [220,221]. Of note, Zarember et al. demonstrated that iron-sequestration by Apo-Lf, released by human polymorphonuclear leukocytes, inhibits *A. fumigatus*, thus suggesting that this mechanism can be active in vivo [222]. Besides a fungistatic effect, in *C. albicans, C. krusei* and *C. neoformans* Lf can exert a fungicidal function through its direct interaction with the fungal cell surface, leading to cell lysis [217,223,224]. Molecular studies on *C. albicans* have shown that Lf was able to impact cell release of potassium ions, cytosolic pH, membrane potential, intracellular ROS production and chromatin condensation, all hallmarks of apoptotic induction [225,226]. In *S. cerevisiae*, Lf was shown to induce cell death in a mitochondrial and caspase-dependent way [227]. Interestingly, a recent study in Lf-null mice, systemically infected by *C. albicans*, has demonstrated that the intravenuos administration of hLf was significantly efficient in: (i) promoting the cleareance of *C. albicans* in different organ tissues; (ii) downregulating proinflammatory cytokines, such as IL-1β, IL-6 and TNF-α, as well as the expression of myeloperoxidase and inducible nitric oxide synthase and (iii) upregulating the antiinflammatory cytokine IL-10, when compared to untreated Lf-null mice [228].

Finally, studies on Lf anti-viral activity against Norovirus, the only eukaryotic virus reported to be possibly involved in IBDs so far, showed that the protein was able to block both viral attachment and replication in murine macrophages [229] and to reduce viral infection in human B cells [230].

**Figure 2:**
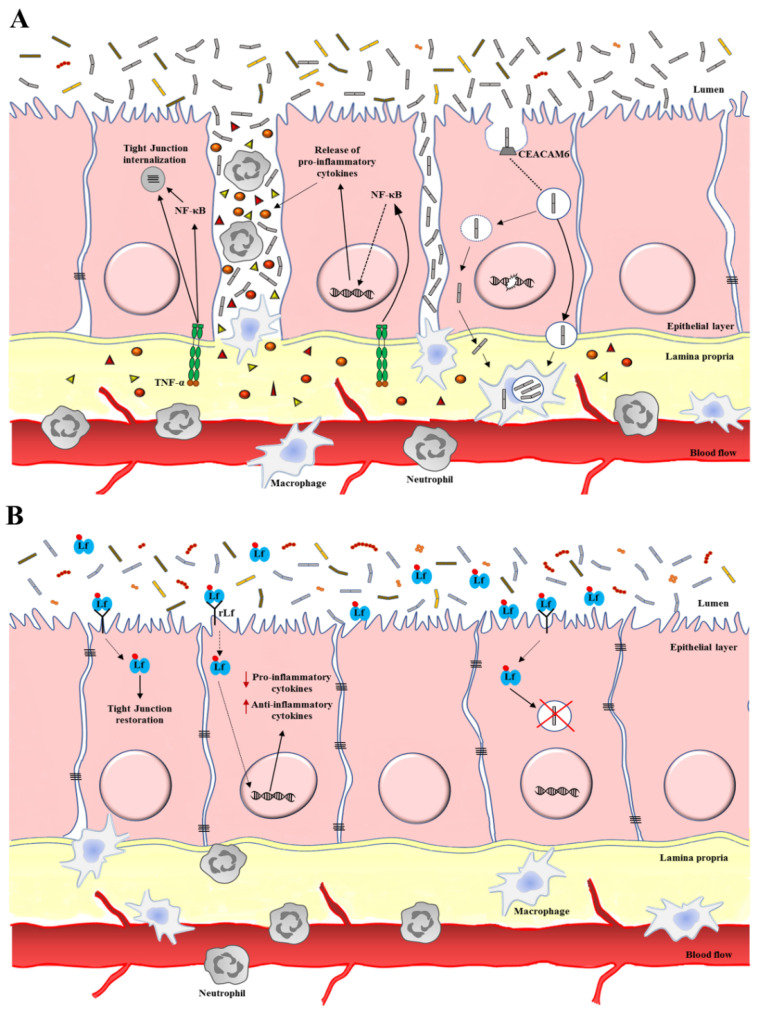
Schematic representation of the role of lactoferrin (Lf) in rebalancing tissue homeostasis in intestinal epithelium affected by an inflammatory bowel disease (IBD). (**A**) In IBD, the intestinal epithelium barrier dysfunction is related to destructive inflammatory stimuli, which, in turn, promote microbial dysbiosis. In this scenario, the intestinal epithelium participates in the recruitment of immune cells through cytokine/chemokine release, mainly mediated by TNF-α and NF-kB pathways, which are also responsible of tight junctions (TJs) internalization. The increased epithelium permeability facilitates cell adhesion and invasion by facultative intracellular pathogens, i.e., AIEC LF82, able to replicate in both enterocytes and macrophages, and to damage cell defense by inducing host DNA damage. (**B**) Effect of Lf treatment on intestinal epithelium. Lf can act as a multi-targeting agent, able to: (i) counteract microbial dysbiosis through its bacteriostatic and bactericidal activities, as well as by interfering with adhesion, invasion and survival of facultative intracellular pathogens; (ii) rebalance immune homeostasis through its ability to modulate/boost immune system and correcting inflammatory imbalance; (iii) restore intestinal barrier integrity by favoring TJs up-regulation and membrane localization. Orange circles and red/yellow triangles represent pro-inflammatory cytokines; red dots attached to Lf represent ferric ions.

Overall, the anti-microbial and antiviral activity of Lf strongly support its application in IBDs as a broad-spectrum compound, able to target entities of different phyla and families involved in the disease pathogenesis, thus counteracting intestinal dysbiosis and protecting the host (Figure 2).

### 3.2. Lactoferrin Anti-Inflammatory Activity against IBDs

As already stated, IBDs are characterized by the persistence of destructive inflammatory processes correlated with microbial dysbiosis and loss of intestinal barrier integrity (IBI) [146]. IBI is a key feature in the health of humans, crucial to prevent the entry of noxious luminal antigens from the intestinal lumen into mucosa and blood circulation [231]. Therefore, the loss of IBI increases mucosal permeability and host susceptibility to pathogens, thus damaging the immune homeostasis as well as exacerbating the inflammatory response [116]. Indeed, IBDs patients generally show an immune over-responsiveness to commensal bacterial antigens, resulting in the activation of many pro-inflammatory cytokines, such as IL-1β, TNF-α, IL-6 and IL-12, related to the increase of neutrophil and pro-inflammatory macrophages [146,232]. In this context, it is observed the loss of some tight junction (TJ) molecules, including claudins, occludin, and zonula occludens, which are involved in the maintenance of intestinal epithelial barrier, thus increasing the host susceptibility to infections, inflammatory disorders and cancer [233,234].

Although the primary evaluation of intestinal barrier integrity is generally diagnosed by endoscopy, fecal biomarkers are becoming a low cost, non-invasive diagnostic method, useful to monitor disease and subsequent relapse. In this respect, the detection of fecal Lf is a very sensitive marker for IBDs, reflecting mucosal inflammation [235]. Indeed, fecal levels of Lf rise quickly due to the influx of neutrophils in intestinal inflammatory sites [231].

The protective function of bLf towards intestinal barrier dysfunctions has been reported in two in vitro studies [236,237]. The study by Hering and colleagues demonstrated that bLf was able to: (i) restore TJ morphometry and inhibit apoptosis in TNF-α challenged human epithelial cell models; (ii) counteract trans-epithelial resistance drop and claudin-8 down-regulation in *Y. enterocolitica* infected human epithelial cell models [236]. The study by Zhao et al. reported that bLf triggers the up-regulation of claudin-1, occludin, and zonula occludens-1 expression, which, in turn, strengthens the barrier function of intestinal cells. Moreover, a decrease of TNF-α mRNA levels and the consequent inhibition of NF-kB pathway were also observed [237].

Even more solid results on bLf anti-inflammatory effects in different IBD animal models have been reported. Togawa et al. demonstrated that in rats with DSS-induced colitis the oral administration of bLf decreased disease severity in a dose-dependent manner, as reflected by the improvement in clinical disease activity index, white blood cell count and hemoglobin concentration, macroscopic and histological scores and reduced myeloperoxidase (MPO) activity, used as a marker for neutrophil infiltration [238]. Reduced inflammation was correlated with a significant reduction in the pro-inflammatory cytokines TNF-α, IL-1β and IL-6, and the simultaneous induction of the anti-inflammatory cytokines IL-4 and IL-10 [238]. Similar effects were reported by Li and colleagues, which pointed out the differential efficacy of apo- and holo-bLf treatments in relieving DSS-induced colitis in BALB/c mice. In particular, the results showed apo-bLf to be more efficient than the holo form in decreasing MPO, IL-1β and TNF-α synthesis, as well as in ameliorating intestinal histology score [239].

Comparable results were obtained in another study of DSS-induced acute colitis in C57Bl/6 mice orally administered with rhLf [240]. Mice treated with rhLf showed a reduction in the appearance of occult blood in the feces and the shortening of the colon when compared with the control group. Also, serum IL-1β levels, number of CD4+ cells, F4/80+ macrophages and TNF-α-producing cells were significantly diminished in rhLf-treated animals [240].

Taken together, these studies suggest that Lf iron-content, rather than Lf source, could be relevant in the efficacy of Lf treatment against colitis. Indeed, as already stated, apo-Lf can exert a potent anti-microbial activity, thus corroborating the anti-inflammatory effects and globally improving intestinal health.

Despite different papers reporting in vitro and in vivo studies, only a single case study on bLf activity against CD was reported [241]. In particular, bLf oral administration, performed after the end of the standard therapy, was able to maintain CD in a remissive state in a 22-year-old male [241].

In this complex context, bLf emerges as an attractive molecule able to target multiple aspects of IBDs, including immune system homeostasis. As matter of fact, bLf has been recently proven to promote the macrophage shift from inflammatory to tolerogenic phenotype [16], this latter being involved in the restoration/maintenance of tissue homeostasis. Moreover, bLf has been extensively reported as a negative regulator for IL-6 synthesis, both in in vitro and in animal model studies [16,35,180,181,242] as well as in clinical trials [38,65,243]. Interestingly, bLf was also found to interfere with STAT3 activation pathways both in IL-6-dependent or -independent mode [23,244].

Although more studies need to be carried out to confirm the beneficial effects of Lf as well as to better understand the underlying molecular mechanisms, it is possible to assert that Lf oral administration can be considered a promising tool for the prevention and treatment of IBDs, through its ability to modulate/boost immune system and to correct inflammatory imbalance (Figure 2).

## 4. Lactoferrin in Colorectal Cancer Prevention

The incidence of CRC is increasing worldwide. Serious IBDs, but also lifestyle and diet, have been suggested to increase the risk of morbidity [245,246]. On the other hand, an important role in preventing cancer is fulfilled by appropriate food consumption, including fruits, vegetables, and fibers [247]. Also Lf can play a role in this respect. So far, hLf, bLf and their derived peptides have been recognized as promising molecules in cancer prevention and treatment. Lf efficiency in inhibiting tumor growth, metastasis and tumor-associated angiogenesis [248,249,250], as well as in advancing chemotherapy [251,252], has been evaluated in different types of tumors, including lung, tongue, esophagus, liver, and colorectal cancers [253,254,255,256]. Molecular mechanisms by which Lf can exert its anticancer activity include induction of apoptosis [15,257], cell cycle arrest [15,258], inhibition of migration and invasiveness [15,249] and immune boosting [15,259]. As already mentioned, the base of Lf anti-cancer specificity could be ascribed to the electrostatic binding, through its cationic N-terminal region, to acidic molecules highly expressed on the surface of cancer cells, including proteoglycans, GAGs and sialic acids [260,261,262].

Besides these relevant mechanisms, the anti-cancer activity of Lf through host immunomodulation has been widely reported, especially in CRC [110,263]. In this regard, it is important to consider that the immune response can trigger both tumorigenic and anti-tumorigenic pathways, depending on the balance between innate and adaptive components, as well as on the subtype and state of the related cancer. Moreover, the tumor microenvironment bears diverse leukocyte populations able to release an assorted repertoire of cytokines [264], cytotoxic mediators, such as ROS, and soluble mediators of cell killing, such as TNF-α and IFNs [264,265]. On this ground, Lf anti-cancer activity through potentiation of the adaptive immune response as well as through shutting down inflammation was postulated [15]. However, although it has been reported that Lf directly modulates anti-cancer immune response through a subclass of leukocytes, natural killer (NK) and cytotoxic T cells [15], the molecular mechanism is still unclear. Several in vitro and in vivo studies have shown that bLf interacts with epithelial cells and immune cells in the mucosa of the intestine thus stimulating production of cytokines, such as IL-10 and IFN-γ [266], and enhancing mucosal immunity [9], NK cell activity and Lymphokine-activate killer activity [267], neutrophil activity and macrophage cytotoxicity [268]. The first observation of bLf as an inhibitor of colon tumorigenesis was in 1995, when the whey fraction of bovine milk was demonstrated to hinder tumor in 1,2 dimethylhydrazine (DMH)-treated rats [269]. Few years later, the Tsuda group demonstrated that bLf was able to inhibit azoxymethane (AOM)-induced colorectal cancer in a rat model [263] and that the glycoprotein reduced the development of aberrant crypt foci and the incidence of intestinal adenocarcinomas through the increase of NK activity [270,271]. BLf was also shown to activate the apoptotic extrinsic pathway through up-regulation of Fas signaling in the colon mucosa of AOM-treated rats [272]. In addition, in BALB/c mice bearing subcutaneous implants of the highly metastatic colon carcinoma 26 (Co 26Lu), oral administration of bLf reduced metastatic colony formation [273] and promoted the increase of CD4+ and CD8+ T cells as well as of asialoGM1+ cells in the blood [273] and in the lymphoid tissues and lamina propria of the small intestine [274].

In 2000, a mechanistic basis for bLf oral administration efficacy for prevention of colon carcinogenesis and metastasis was given by Tsuda group, which found that, upon bLf treatment, murine intestinal mucosa produced the pro-inflammatory cytokine IL-18 and caspase-1 [56] as well as IFN-γ [275], and activated T and NK cells, thus providing elevated systemic protection against carcinogenesis, metastasis and angiogenesis [276]. Interestingly, bLf was demonstrated to increase NK activity also in IL-18 KO mice [277], where bLf also increased expression of IFN-α and IFN-β (type I IFNs) in Payer’s patches and mesenteric lymph nodes [278]. Consistent with this, bLf can activate more than one effector pathway; which ones are responsive to activation depend on the physiology of the gastrointestinal tract. In addition, in the absence of the IFNγ/IL-18 effector pathway, bLf is able to exert anti-carcinogenesis activity by activating a second effector avenue, the IFN-α/IL-7 pathway [279]. Also, bLf induced the expression of IL-15 by peritoneal macrophages in vitro but the specific role remains to be elucidated [279]. Recently, it has been demonstrated that DMH/DSS-induced CRC is inhibited by the oral administration of liposomal bovine lactoferrin (LbLf) in rats [255]. LbLf-fed rats harbored significantly fewer aberrant cryptic foci, adenomas and adenocarcinomas of the colon, with respect to the control group, and showed decreased cancer cell growth and TNF-α mRNA expression [255].

In humans, Lf application for cancer prevention is almost impracticable for most of the tumors. A randomized clinical trial was conducted in 2009 to evaluate bLf efficacy on the growth of colorectal benign tumors, called polyps, in order to prevent transformation into cancerous cells [280]. This study was performed on patients, aged 40 to 75 years, with polyps of 25 mm diameter and likely to bear pre-cancerous lesions, at the National Center Hospital, Tokyo, Japan. Participants were assigned to receive placebo, 1.5 or 3.0 g of orally administered bLf daily for 12 months. The group treated with the higher bLf dose presented a significant reduction in colorectal adenomatous polyps size compared to placebo and lower dose groups [280]. The same research group later reported a correlation between immune cell parameters and colorectal polyps size observed in the Tokyo trial. The connection between higher immune activity and suppression of benign tumors sizes was found in increased NK cell activity, numbers of CD4+ and CD161+ cells and high serum hLF levels, but also in decreased inflammatory stimuli, consistent with the observed reduction of neutrophils and increased numbers of S100A8+ cells in the polyps [281].

Taken together, these findings suggest that protracted administration to animals and daily intake for one year by human patients is efficient in counteracting colon cancer development, mainly by triggering an immune response. Last, but not least, in all of the animal and human studies conducted to date, ingestion of bLf has been shown to be toxicologically safe.

## 5. Conclusions

Inflammatory bowel disease is a multifactorial pathology, primarily linked to an aberrant immune response to gut commensal flora, mainly bacteria, in a genetically susceptible host. Disruption of this normal microbial-host communication network has deleterious consequences for the host and it is also associated to higher risk for colorectal cancer. Despite new therapies are aimed at both achieving remission and preventing disease flares, no efficient treatments have been found yet. In this scenario, alternative options are constantly sought by patients, including dietary modifications, prebiotics and probiotics as well as food supplements, and, among these latter, lactoferrin can be considered a very promising approach.

As a nutraceutical, Lf activity relies on its physico-chemical properties, which are, in turn, dependent on the source quality and manufacturing procedures. The main source of Lf remains milk, whose composition strongly correlates with the breed and well-being of the animals, as well as with environmental factors, such as season and maternal diet. Hence, it is imperative to evaluate the physico-chemical features of purified Lf, in terms of iron content, glycosylation pattern, integrity, as well as presence of contaminants, such as lactoperoxidase or LPS [21]. In fact, all these factors can influence most of Lf functions: the anti-microbial activity is impaired by elevated iron content by hindering the iron-dependent bacteriostatic capacity of the protein; the anti-inflammatory activity is influenced by contamination with LPS, which can, in turn, activate the pro-inflammatory response; the ability of Lf to enter into the cell nucleus, and regulate gene expression, is dependent on protein integrity [21]. These concerns have been also correlated with the conflicting results reported by some functional studies on Lf, in particular for its anti-inflammatory activity, more prone to be influenced by the quality of different commercial or self-produced Lf preparations as well as by the biological system taken into consideration [14]. For instance, despite numerous studies on Lf-mediated IL-6 down-regulation, only few reported an induction of this cytokine in murine peritoneal macrophages via both a TLR-4 independent and dependent pathway [282,283]. Moreover, bLf was reported to induce IL-6 in monocyte-derived dendritic cells (MD-DCs), whereas no up-regulation was observed after MD-DCs differentiation [284]. These studies have demonstrated, once again, the dichotomy of Lf, which can exert opposite effects depending on the biological system it acts upon.

Lf bioavailability remains a key issue to be solved. Dietary Lf, included in both natural and supplemented foods, incurs in both enzymatic digestion and physical barriers of the gastrointestinal mucosa, which contribute to its poor permeability across the gut epithelium. To overcame this drawback, two strategies have been carried out: the administration of purified Lf (usually bLf) at higher dosage and, more recently, the development of new formulations of Lf aimed at improving its bioavailability [285]. In this regard, despite several studies have proven Lf to be safe and well-tolerated even at high dosages [286,287], Nguyen et al. reported that the effect of bLf on the inflammatory balance of both intestinal epithelial cells and immature pig intestine is strictly dose-dependent. Indeed, low doses of bLf (0.1–1 g/L in the drinking water) triggered a beneficial effect through the regulation of proteins supporting cell homeostasis, whereas high doses of bLf (10 g/L in the drinking water) induced the apoptotic process and an inflammatory response in both in vitro and in vivo models [288].

**Figure 3:**
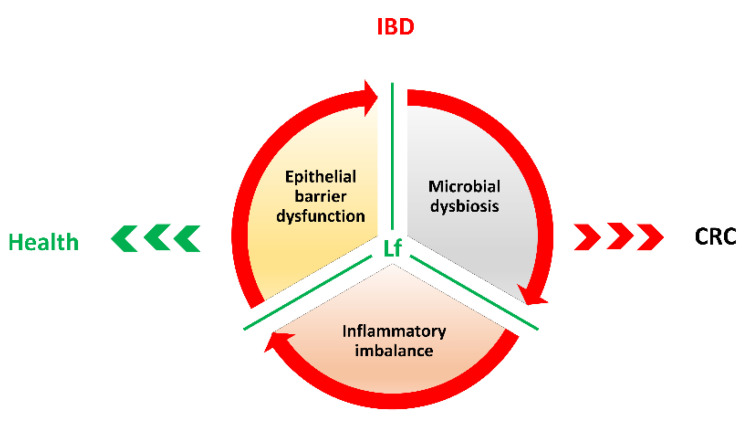
Summary diagram on Lf multi-targeting activities (green lines) counteracting, and potentially reverting, the unsafe vicious cycle established by microbial dysbiosis, inflammatory imbalance and intestinal barrier dysfunction in IBDs. CRC: colorectal cancer.

Regarding IBDs, and related-CRC, Lf bioavailability should not be considered a limiting issue, as is true for the treatment of peripheral pathologies, which require the delivery of Lf through systemic or lymphatic circulation. As matter of fact, the large unabsorbed portion of the glycoprotein can easily exert both anti-microbial and anti-inflammatory actions by directly interacting with the intestinal mucosa, thus reverting microbial dysbiosis, inflammatory imbalance and epithelial barrier dysfunction (Figure 3).

As emerging from this review, Lf acts by: (i) boosting the host immune response; (ii) inhibiting destructive pro-inflammatory processes; (iii) strengthening of intestinal barrier and (iv) antagonizing the action of pathogens. Such mechanisms have been mainly investigated in both in vitro and animal models, however, conclusive clinical trials are strongly needed, as experimental models could be very far from the actual biological complexity and diversity of human biome. In this respect, controversial results on the efficacy of Lf against *H. pylori* gastric infection in in vitro, in vivo and clinical studies have been reported. In particular, after the pioneering study by Miehlke and colleagues [289], who demonstrated the in vitro bactericidal activity of rhLf against 13 clinical isolates of *H. pylori*, both bLf and rhLf were shown to promote bacterial growth and gastric inflammation in mice [290]. In the same field, inconclusive results have been obtained so far in humans, with two open randomized trials showing the efficacy [291,292] and one the inefficacy [293] of bLf in improving eradication rates of *H. pylori* when applied in combination therapies. Moreover, despite the abovementioned studies proving the efficacy of Lf on animal models of colitis, the failure of bLf in decreasing risk of sepsis and necrotizing enterocolitis (NEC) in preterm infants was recently reported [294]. The ELFIN trial disclaimed previous studies, each carried out on a smaller number of patients, which reported the significant protective effects of bLf supplementation against NEC onset in very low-birth-weight neonates [295,296,297,298,299]. Hence, only clinical trials will confirm or disclaim the efficacy and the potential application of Lf in IBDs.

## Figures and Tables

**Table 1:** Lactoferrin concentration in milk and dairy products from different species.

Milk or Dairy Product		Lactoferrin mg/mL	Detection Assay	Reference
Cow Milk	Raw	0.020–0.200	Radial Immunodiffusion	[83]
		0.157 ± 0.007	ELISA	[84]
		0.182 (RSD% ≤ 5.5)	Immunosensors	[85]
		188.4 ± 13.2	Reversed-phase HPLC	[86]
	Pasteurized	0.174 ± 0.017	ELISA	[84]
	UHT	0.018 (RSD% ≤ 5.5)	Immunosensors	[85]
Buffalo milk	Raw	0.232 (RSD% ≤ 5.5)	Immunosensors	[85]
Goat Milk	Raw	20–200	Radial Immunodiffusion	[83]
		0.125 ± 0.020	Reversed-phase HPLC	[87]
		0.042 ± 0.008	ELISA	[88]
		927.3 ± 52.1	Reversed-phase HPLC	[86]
	Pasteurized	0.105 ± 0.015	Reversed-phase HPLC	[87]
	Partially skimmed	0.017 (RSD% ≤ 5.5)	Immunosensors	[85]
Sheep Milk	Raw	0.260 ± 0.050	Radial Immunodiffusion	[89]
		0.466 ± 0.023	Reversed-phase HPLC	[86]
Camel Milk	Raw	0.229 ± 0.135	Radial Immunodiffusion	[90]
Cheese	Swiss type	1.112 ± 0.111 mg/g	ELISA	[84]
	semi-hard	1.143 ± 0.118 mg/g	ELISA	[84]
	soft	0.680 ± 0.015 mg/g	ELISA	[84]
	Feta	0.272 ± 0.024	Reversed-phase HPLC	[86]

**Table 2:** Maximum intended use levels of bovine lactoferrin in supplemented foods [96,97].

Food	BLf Maximum Level (EU)	BLf Maximum Level (USA)
Infant formulae—powder	30–770 mg/100 g	100 mg/100 g
Infant formulae—ready-to-feed	4–100 mg/100 mL	13 mg/100 mL
Infant formulae—liquid concentrate	26 mg/100 mL	-
Milk beverages	50–200 mg/100 g	100 mg/100 g
Powdered milk	300 mg/100 g	400 mg/100 g
Ice creams	130 mg/100 g	200 mg/100 g
Sherbets	-	200 mg/100 g
Yogurt	80 mg/100 g	100 mg/100 g
Chewing gum	30 mg/g	30 mg/g
Processed cereal food	670 mg/100 g	-
Product based on cheese	2000 mg/100 g	-
Non alcoholic drinks	120 mg/100 g	-
Cakes and pastries	1000 mg/100 g	-
Candies	7 mg/g	-
